# Quantum Embedding of Non-Local Quantum Many-Body Interactions in an Prototypal Anti-Tumor Vaccine Metalloprotein on Near-Term Quantum Computing Hardware

**DOI:** 10.3390/ijms26041550

**Published:** 2025-02-12

**Authors:** Elena Chachkarova, Terence Tse, Yordan Yordanov, Yao Wei, Cedric Weber

**Affiliations:** 1 Theory and Simulation of Condensed Matter (TSCM), King’s College London, The Strand, London WC2R 2LS, UK; elena.chachkarova@kcl.ac.uk (E.C.); yao.wei@kcl.ac.uk (Y.W.); 2 Cavendish Laboratory, Alumni—Cambridge University, Cambridge CB3 0HE, UK; yy387@cam.ac.uk

**Keywords:** hemocyanin, molecular modeling, metalloproteins, variational quantum eigensolver, strongly correlated quantum systems, spin–spin correlation, quantum embedding, quantum benchmarking

## Abstract

The world obeys quantum physics and quantum computing presents an alternative way to map physical problems to systems that follow the same laws. Such computation fundamentally constitutes a better way to understand the most challenging quantum problems. One such problem is the accurate simulation of highly correlated quantum systems. Still, modern-day quantum hardware has limitations and only allows for the modeling of simple systems. Here, we present for the first time a quantum computer model simulation of a complex hemocyanin molecule, which is an important respiratory protein involved in various physiological processes and is also used as a key component in therapeutic vaccines for cancer. To characterize the mechanism by which hemocyanin transports oxygen, variational quantum eigensolver (VQE) and quantum embedding methods are used in the context of dynamic mean field theory to solve the Anderson impurity model (AIM). Finally, it is concluded that the magnetic structure of hemocyanin is largely influenced by the many-body correction and that the computational effort for solving correlated electron systems could be substantially reduced with the introduction of quantum computing algorithms. We encourage the use of the Hamiltonian systems presented in this paper as a benchmark for testing quantum computing algorithms’ efficiency for chemistry applications.

## 1. Introduction

Quantum computing introduced a new approach to solving challenging computational problems that are difficult for classical computers [1,2]. Development in the field of quantum algorithms, as well as quantum hardware, has captured the interest of many large companies and academic researchers. These efforts have paved the way for applications that leverage the unique capabilities of quantum devices to potentially surpass the best-known classical algorithms in various domains. Notable examples include integer factorization [3,4], financial portfolio optimization [5,6], fraud detection [7], and the simulation of complex quantum systems [1,2,8,9,10,11]. To further underscore the significance of this research, it is valuable to consider the global economic implications of advancements in the quantum computing manufacturing sector. For instance, in the realm of drug discovery, quantum computing is projected to reduce research and development costs by approximately USD 10 billion annually by 2030. More broadly, the quantum computing industry is expected to generate from USD 450 billion to USD 850 billion in economic value by 2040, supporting a market estimated to be worth USD 90 billion to USD 170 billion for the development of quantum hardware and software [12].

Despite rapid progress, current quantum hardware, often referred to as noisy intermediate-scale quantum (NISQ) devices, is subject to limitations such as qubit noise and decoherence. These issues restrict the accessible problem sizes and complexities. Qubits typically need to be maintained at cryogenic temperatures, often just a few millikelvins above absolute zero, to ensure stability and minimize thermal noise. Furthermore, the development of quantum hardware faces additional challenges due to the need for cryogenic cooling systems to maintain ultra-low temperatures and ensure precise temperature control while minimizing thermal noise and energy dissipation. Meanwhile, efforts to mitigate noise, including various error mitigation strategies [13,14] and error correction protocols [15,16], are progressing at a good pace. The ongoing improvements in quantum hardware stability, fault tolerance strategies [17], and device scalability [18,19,20,21] indicate that quantum computing may soon reach a point where it can offer advantages over classical methods in solving large parameter–space problems.

Quantum chemistry is a focus area where quantum computing can overcome many limitations of classical computer algorithms that become restricted due to the high degree of complexity of underlying physical processes [22,23,24]. For instance, calculating molecular energies within chemical accuracy requires exponential scaling of computational resources, and large systems are primarily tackled by costly experimental methods. The introduction of robust quantum algorithms to model and solve electron systems could unleash a new era of material discovery and provide insights into puzzling challenges like room temperature superconductivity and strongly correlated materials modeling. Computation that employs quantum principles is the most fundamental way to determine material characteristics and allows us to map electron states directly onto qubit quantum states, which could, in many cases, be the most optimal tool for understanding these systems.

**Figure 1:**
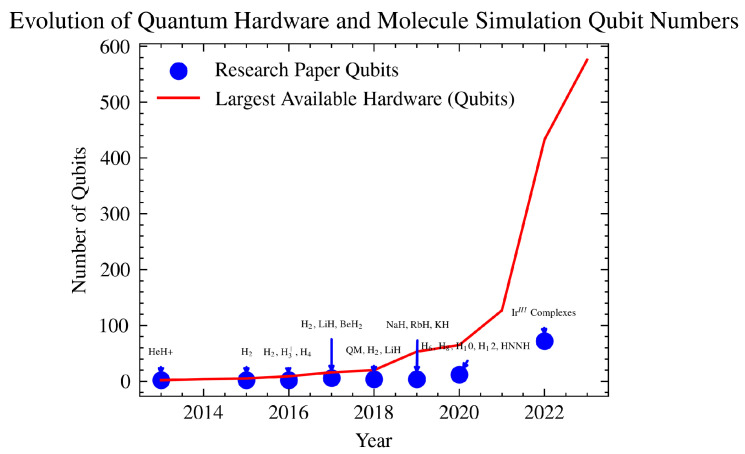
Progression of quantum computing technology from 2013 to 2023, highlighting two key trends: the rapid increase in the maximum number of qubits available in quantum hardware (shown in red) and the highest number of qubits used in significant quantum computing-based molecule simulation research papers each year (shown in blue). Each point in the research data series is labelled with the specific quantum system studied, providing insight into the scale of quantum experiments and their corresponding hardware capabilities over time. Due to the accumulation of noise, the size of the molecules studied is not increasing as rapidly as the advancements in hardware capacity [39].

So far, quantum hardware has primarily been used to explore the ground state properties of relatively simple molecules with a small number of electrons, such as the water molecule (H_2_O) [25,26] and hydrogen molecules [27]. This focus is due to the increased noise associated with using more qubits and applying entanglement. Recently, there has been growing interest in addressing the challenges associated with strongly correlated electron systems, not only for ground state energy estimation but also for other system characteristics. Notably, work by Microsoft Azure Quantum [28] has introduced an end-to-end comprehensive method for simulating strongly correlated molecular systems. This approach leverages a hybrid classical-quantum method and incorporates the classical shadows technique [29] for property measurement, all hosted on the Microsoft Azure platform. This paper complements this work by aiming to measure a complex system and its properties using quantum hardware. To expand the context, Figure 1 illustrates the evolution of quantum computer-based materials modeling research across various molecules of different sizes and complexities based on data from Table 1. To estimate the ground state energy of these molecules, the VQE method has been employed [30,31,32,33,34]. VQE is a hybrid algorithm that uses both classical computers and quantum computers to estimate the ground state energy of a Hamiltonian. It uses the Rayliegh–Ritz variational principle [35] and can model complex ground state wavefunctions in a polynomial time using an ansatz defined by a set of parameters that constitutes a trial measurement, which is fed into a classical optimizer that iteratively updates the ansatz parameters to reduce the energy until it converges. The method is sensitive to the form of the ansatz, topology of the hardware, initial state, and more, but tailoring those provides flexibility and investigations into VQE simulations can lead to a recipe for the most optimal problem-specific VQE setup. Here, for the first time, we present the hardware VQE simulation of the complex hemocyanin molecule [36] modeled by AIM [37] on the IBM Quantum platform [38].

The interest in hemocyanin (abbreviated as Hc) came from its fascinating properties and applications; for instance, Hc could be a structural part of metallodrug design, but that requires a full understanding of its complex structure, which remains challenging to model even with modern-day conventional methods [57]. Hemocyanins are a key component in therapeutic vaccines for cancer due to their useful carrier qualities [58]. Hemocyanins are also used as nonspecific immunostimulants for the treatment of superficial bladder cancer (SBC), for which these glycoproteins have demonstrated several advantages over more standard immunotherapeutic procedures [59]. Furthermore, the hemocyanin known as keyhole limpet hemocyanin (KLH) has been applied in various in vitro and preclinical studies to determine its effectiveness against other cancers, such as Barrett’s adenocarcinoma [60], pancreatic, breast, and prostate cancer [61], and melanoma [62,63]. In nature, Hc is a protein that transports oxygen in the blood of some invertebrate animals and is more resilient to the surrounding environment than haemoglobin. Although less efficient, Hc can be fully functional in low-oxygen environments and cold as well as hot temperatures of up to 90 °C. Modeling the formation of the oxygenated state of Hc remains a challenge, as the binding of the O_2_ is a spin-forbidden transition. To model this system, a classic dynamical mean field theory (DMFT) model [64,65,66] would be insufficient as it would treat Cu atoms separately, and it is believed there is a superexchange pathway between the Cu d-orbitals and intermediate O p-orbitals. Hence, a multi-site AIM is needed to probe the correlated sites directly [67,68]. The spin-forbidden multi-site binding transition in Hc represents a challenging but insightful model for quantum systems and, as such, could find its application as a template for multi-qubit entanglement strategies. By leveraging the multi-site correlated interactions observed in Hc, metalloenzyme-inspired AIM methods can potentially inform the design of molecular systems capable of supporting quantum entanglement. As mentioned in [69], the redox-active nature of such metalloenzyme-like frameworks can serve as a foundation for designing quantum chemical approaches that simulate electron transfer and spin transitions, which are critical for quantum computing and sensing applications. Metal-organic frameworks (MOFs), particularly those incorporating metallo-porphyrin structures like vanadyl porphyrin [70], offer a modular and tunable framework for coupling electronic and magnetic states at the nanoscale. Their ability to localize and control interactions within 2D nanosheets aligns with the requirements for quantum technologies, such as qubit arrays or quantum sensors. Moreover, the coupling of MOFs with superconducting resonators demonstrates their suitability for integration with existing quantum hardware platforms, paving the way for hybrid quantum technologies.

**Figure 2:**
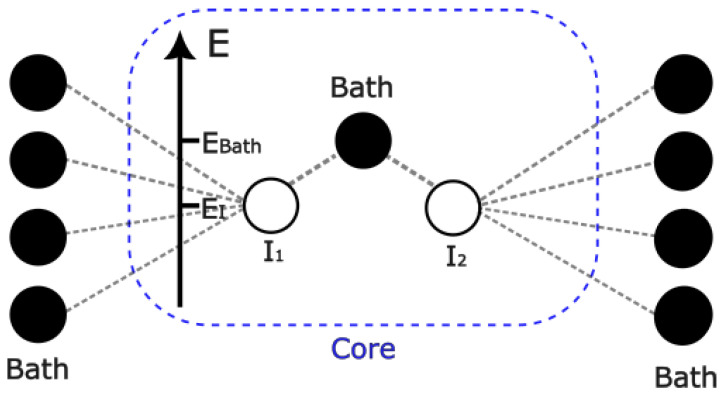
Diagram illustrating the topology of the Cu_2_O model, showing the difference in energy levels between bath and impurity sites in the core. Here, the impurities and bath represent the Cu and O atom sites, respectively.

Work has been carried out for the Cu_2_O cluster model, as shown in the diagram in Figure 2. It should be noted that the diagram is for illustrative purposes only, and the topology shown can be extended to all-to-all connected models. It can be derived that the Heisenberg antiferromagnetic coupling *J* can be expressed as follows:

(1) $$J=4\frac{t_{pd}^{4}}{\Delta^{2}}\left[\frac{1}{U_{d}}+\frac{1}{\Delta +U_{p}/2}\right],$$

where Δ, $t_{pd}$, $U_{p}$, and $U_{d}$ represent the charge-transfer energy, Cu-O hopping parameter, and on-site Coulomb energies at the O and Cu sites, respectively [71]. Hence, we already have an example of an effective model obtained via embedding, as the bath sites here represent the O_2_ bridge of hemocyanin rather than the oxygen sites of Cu_2_O. While most molecular systems are well-captured by density functional theory (DFT) (+U) methods [72], some generate large collections of quantum states and, therefore, would be captured better through quantum computing. One such case is transition metal proteins at certain temperatures, where extensive collections of near-degenerate states exist. These usually occur in magnetic systems near a low-to-high spin transition driven by Hund’s coupling. Another case is systems with a non-local exchange, such as the superexchange across bridges of transition metal centers. For the former case, work has been carried out to investigate such systems using quantum methods, with the notable example of iron porphyrin [73].

Readers are strongly encouraged to read Section 4 before Section 2 to understand the transformation of a molecular Hamiltonian into a modeled AIM Hamiltonian and the construction of VQE simulation and its components. Section 2 presents the outcomes of VQE simulation runs for different VQE setups, and Section 3 expands on the meaning of the results and proposes ideas to further the research. Finally, in Section 5, we present our conclusions along with suggestions for future work.

## 2. Results

### 2.1. Ground-State Energy Measurement

**Figure 3:**
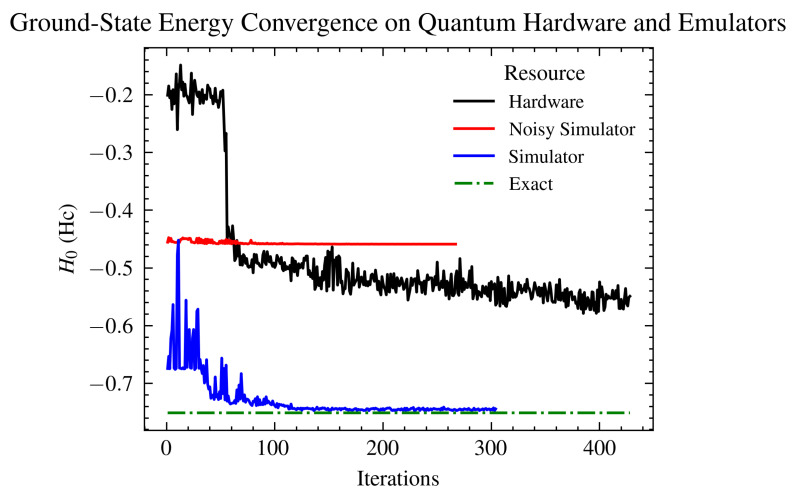
Ground-state energy estimates in $E_{h}$ for a 6-qubit hemocyanin model using VQE simulations on both the IBM Perth hardware and the IBM QASM simulator. Results from the noiseless simulator converge to the expected outcome within 120 iterations, demonstrating high accuracy. Conversely, the hardware execution reaches a barren plateau [74], exhibiting only minimal, continuous decline. Additionally, the noisy simulator, employing a noise model replicated from the actual hardware, fails to identify any feasible solution attributable to the absence of error mitigation and correction techniques. In each instance, the setup employed a generalized UCCSD ansatz. The exact value is provided by [75].

**Figure 4:**
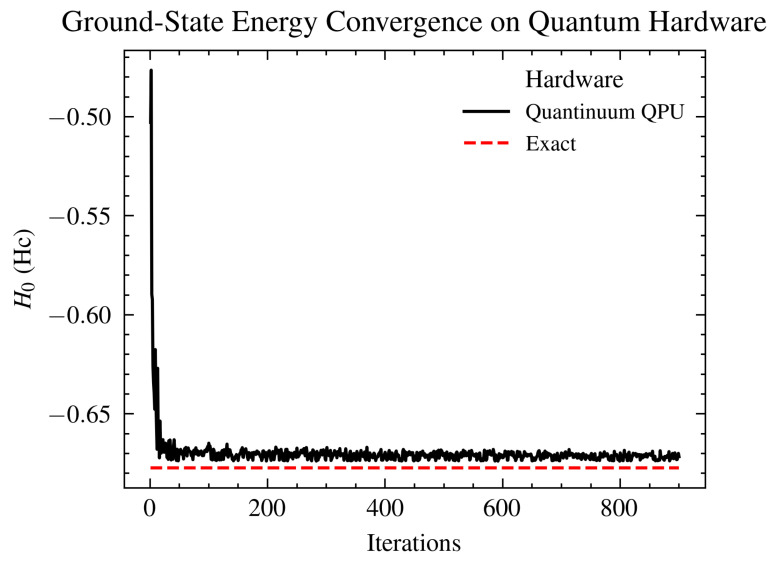
Ground-state energy estimates in $E_{h}$ for a 6-qubit hemocyanin model from VQE simulation on Quantinuum hardware using the Microsoft Azure Quantum platform [28]. The VQE simulation employed a generalized UCCSD ansatz and SPSA [76] optimizer with maximum iterations set to 10,000. The simulation shows a clear convergence to the exact result within an accuracy of ≈0.485% to the exact value [75].

We implemented the qiskit VQE algorithm on 6-, 8-, 10-, 14-, and 16-qubit Hc AIM model Hamiltonians on an IBM quantum simulator with and without noise and on IBM and Quantinuum quantum hardware. Results in the paper present data from 6- and 14-qubit runs to reduce duplication and access extra runs, where Hamiltonians consult with the code. In all cases, the simulations employed a generalized UCCSD ansatz. Figure 3 shows a comparison between the converged values as well as the exact value. The noisy simulator (noise mapped from ibm_Casablanca IBM hardware) VQE run did not show convergence without any error mitigation techniques. The noise, in this case, is too large compared to the changes in the energy estimates from the VQE excitations, and the simulation fails to progress. Figure 4 shows clear convergence on the Quantinuum quantum processing unit (QPU) within 0.485% accuracy of the ground state energy estimate.

### 2.2. Ansatz Construction

**Figure 5:**
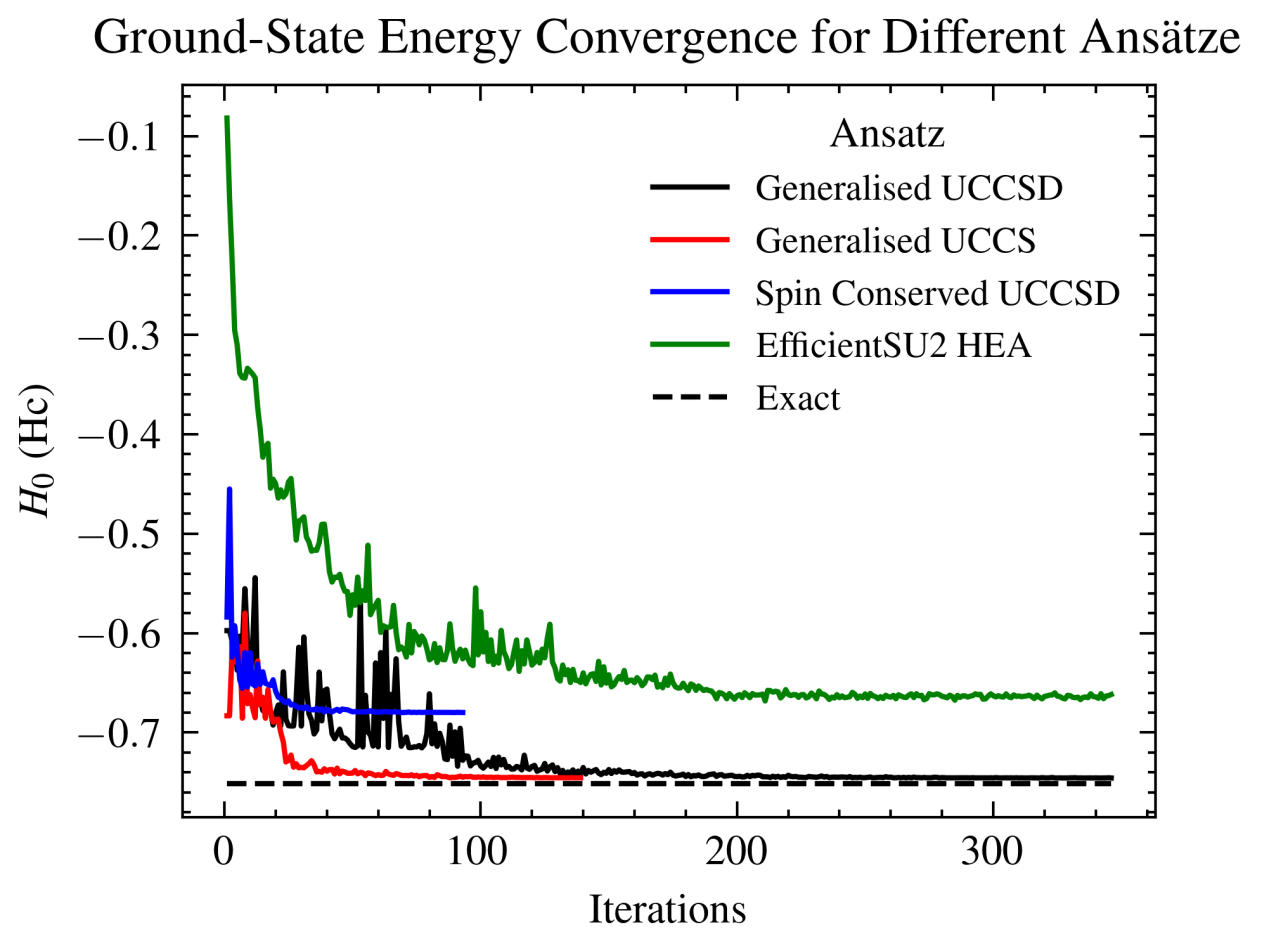
Comparison of ground-state energy estimates in $E_{h}$ derived from the incorporation of additional interactions in the UCC ansatz states, labeled according to ansatz construction parameters. The generalized UCCS ansatz identifies a feasible solution most rapidly. Additionally, the inclusion of second-order excitations also results in correct convergence. In contrast, the EfficientSU2 HEA ansatz converges to a barren plateau. The spin-conserved UCCSD ansatz achieves convergence swiftly, probably due to the restricted number of pool operators, yet it also plateaus.

Choosing the most optimal ansatz for a VQE simulation is of great importance and can considerably change the final result. In Figure 5, we present a plot of the model Hc Hamiltonian ground-state energy estimation on an IBM simulator for different forms of the ansatz: generalized UCC-S/SD-type ansatz, spin conserving UCCSD ansatz, and hardware efficient ansatz, all compared to the exact value. As expected, the output of the simulation runs follows different convergences, which testifies to the strong sensitivity of VQE to the ansatz.

### 2.3. Noise Models

**Figure 6:**
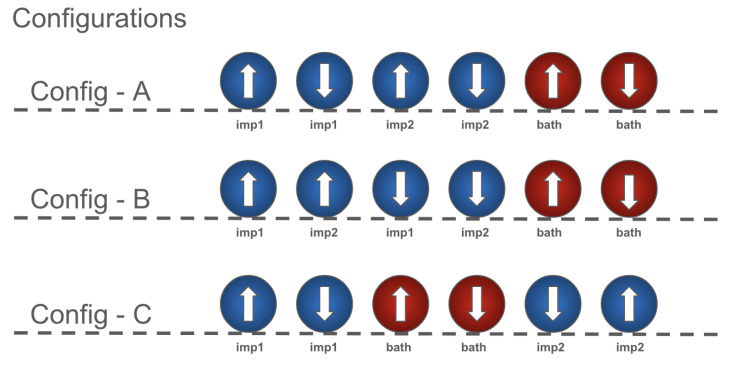
Qubit mappings of the Anderson impurity model of the Hc molecule with two impurities (blue spheres) and one bath site (red spheres). The configurations mark different ordering of the sites onto the underlying hardware with the same coupling map, referencing Figure 7.

**Figure 7:**
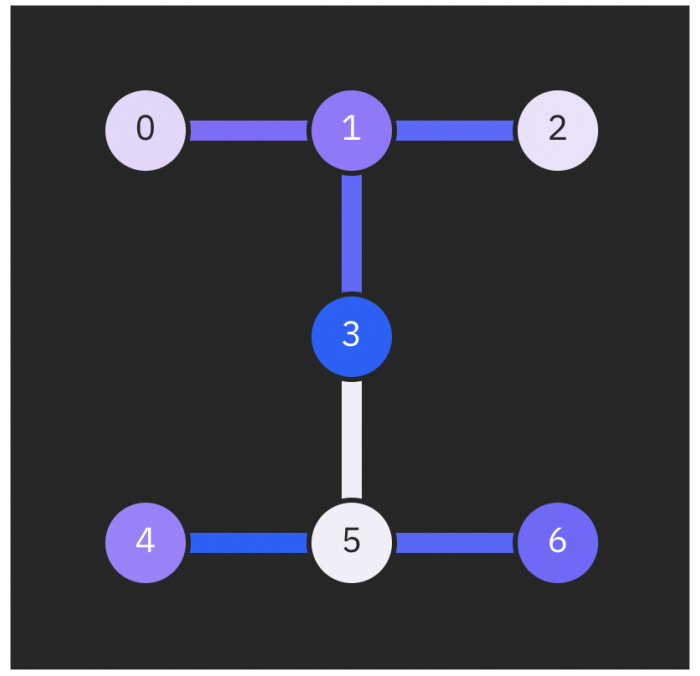
Illustration of the coupling map of IBM_Oslo quantum hardware, delineating which qubits have direct connectivity with each other. Qubits that are not directly connected are also indicated, providing a comprehensive view of the inter-qubit relationships within this quantum hardware configuration.

**Figure 8:**
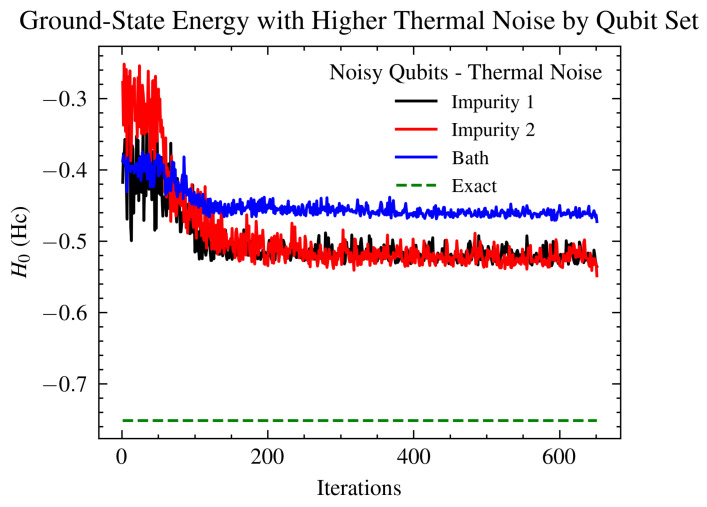
Comparison of ground-state energy estimates in $E_{h}$ from various 6-qubit VQE simulation setups utilizing the generalized UCCSD ansatz, Hamiltonian Config−A, and specific VQE parameters. The simulations were subjected to $T_{1}/T_{2}$ thermal relaxation noise and selectively applied to three different sets of qubits: impurity 1 (qubits 0 and 1), impurity 2 (qubits 2 and 3), and the bath (qubits 4 and 5). Consistent with expectations due to the correlation between impurity 1 and impurity 2 sites, noise impacting either set results in similar values for the ground state energy. Conversely, noise affecting the bath sites demonstrates more significant detrimental effects on the system’s performance. Figure A1 shows the same setup for different level of noise, for further information.

**Figure 9:**
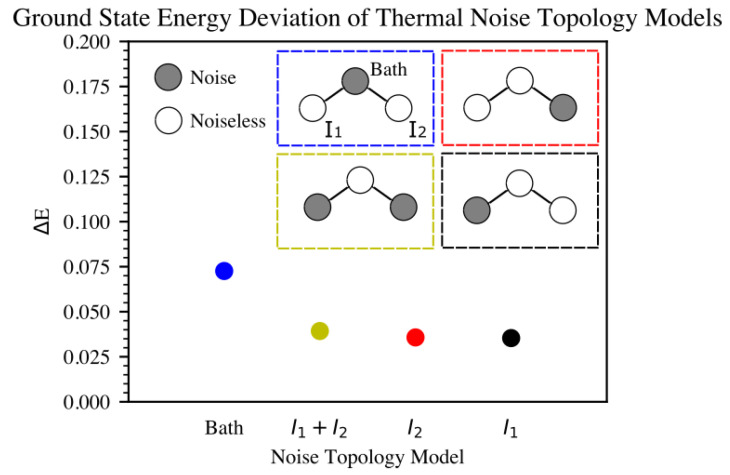
Comparison diagram of ground-state energy deviation in $E_{h}$ from various 6-qubit VQE simulation setups utilizing the generalized UCCSD ansatz, Hamiltonian Config−A, and specific VQE parameters. The simulations were subjected to $T_{1}/T_{2}$ thermal relaxation noise and selectively applied to four different sets of qubits: impurity 1 (qubits 0 and 1), impurity 2 (qubits 2 and 3), impurity 1 and impurity 2 (qubits 0, 1, 2, and 3), and the bath (qubits 4 and 5). Consistent with expectations due to the correlation between impurity 1 and impurity 2 sites, noise impacting either or even both sets results in similar values for the ground state energy. Conversely, noise affecting the bath sites demonstrates more significant detrimental effects on the system’s performance.

Real quantum devices have many sources of error. Previously, we investigated the effect of mapping the total noise from a real quantum computer onto a simulator. This involves mapping the error rates on instructions that are determined by gate times and qubit $T_{1}$ and $T_{2}$ values, where $T_{1}$ is an energy relaxation time (the time it takes for the excited |1〉 state to decay toward the ground state |0〉) and $T_{2}$ is a dephasing time constant. Here, we add custom noise to only specific qubits with the aim of better understanding which parts of the simulation cause higher instabilities. We applied $T_{1}/T_{2}$ thermal relaxation noise to the qubits that represent impurity 1 and impurity 2, both impurities sites (1 and 2), and the bath site, in turn. The Hamiltonian was mapped into the Config−A configuration, as shown in Figure 6 in connection to Figure 7, in all four cases. Results from the simulations are shown in Figure 8 and Figure 9.

#### 2.3.1. Definitions of $T_{1}$ and $T_{2}$

$T_{1}$ and $T_{2}$ values for the qubits are generated randomly based on a normal distribution, where $T_{1}$ averages 50 microseconds with a standard deviation of 10 microseconds, and $T_{2}$ averages 70 microseconds with a standard deviation of 10 microseconds.

#### 2.3.2. Truncation of $T_{2}$ Values

It is physically inferred for $T_{2}$ to be at most twice $T_{1}$ (since $T_{2}$ involves both relaxation and other dephasing effects), thus $T_{2}$ values are truncated to be no more than twice their corresponding $T_{1}$ values for each qubit.

#### 2.3.3. Quantum Gate and Measurement Times

Instruction times are the durations for each type of quantum operation in nanoseconds. For example, the U2 gate, which involves a single X90 pulse, takes 50 ns, and the U3 gate, which involves two X90 pulses, takes 100 ns. Longer operations like measurement and reset are set to 1000 ns (1 microsecond).

#### 2.3.4. Creation of Quantum Errors

Using the thermal_relaxation_error(t1, t2, time) function from Qiskit’s Aer module to simulate quantum noise [77], thermal relaxation errors are created for each quantum operation based on their durations and the $T_{1}$ and $T_{2}$ values of each qubit.

#### 2.3.5. Noise Model Analysis

System noise for a 6-qubit Hc Hamiltonian: By utilizing the cluster Hamiltonian (Equation (2)) derived from the one-band and three-band Hubbard models of cuprates [71], we can map the hemocyanin Hamiltonian to compute the Heisenberg exchange coupling, *J*, with the aim of examining how the system varies with noise introduced by different parameters, as described in Equation (4), where site labels i=1,2 are for impurity 1 and 2 and i=3 for the bath.

(2) $$\begin{array}{cc} \hat{H}=\frac{\Delta }{2}\left(n_{3}-n_{1}-n_{2}\right)-t_{pd}\sum_{i,\sigma }\left(d_{i\sigma }^{†}p_{\sigma }+H.c.\right) & \\ \; & +U_{d}\left(n_{1↑}n_{1↓}+n_{2↑}n_{2↓}\right)+U_{p}n_{3↑}n_{3↓} \end{array},$$

where $n_{d}=d_{i\sigma }^{†}d_{i\sigma }$ (for i=1,2), $n_{3\sigma }=p_{\sigma }^{†}p_{\sigma }$, and 

(3) $$n_{i}=n_{i↑}+n_{i↓}+2t_{pd}\left(\frac{1}{J}\right)\left(1-\frac{1}{2}\right)i\sigma .$$

Here, $d_{i\sigma }^{†}$ creates a hole with a *z* component of spin σ=±1 in the impurities site *i* (i=1,2), and $p^{†}$ creates a hole with a *z* component of spin σ=±1 in the bath site. The charge-transfer energy is Δ, $U_{p}$ and $U_{d}$ are the on-site Coulomb energies at the bath and impurities sites, respectively, and $t_{pd}$ is the strength of hopping between neighboring bath and impurities sites.

(4) $$J=\frac{4t_{pd}^{4}}{\Delta^{2}}\left(\frac{1}{U_{d}}+\frac{1}{\Delta }\right),$$

(5) $$E_{bath}\approx -0.0633E_{h},$$

(6) $$E_{imp1}\approx -0.2842E_{h},$$

(7) $$E_{imp2}\approx -0.2633E_{h},$$

where $E_{bath}$, $E_{imp1}$, and $E_{imp2}$ are the bath and impurities on-site energies, respectively.

(8) $$\Delta =E_{bath}-\frac{E_{imp1}+E_{imp2}}{2}\approx 0.2104E_{h}.$$

(9) $$U_{d}\approx 0.2934E_{h};\;t_{pd}\approx 0.0578E_{h};\;U_{p}\approx 0E_{h}.$$

We assume noisy $\Delta^{\prime }$ and $t_{pd}^{\prime }$ of the following form:

(10) $$\Delta^{\prime }=\Delta +\mathrm{GaussianNoise}\times \mathrm{Amplitude},$$

(11) $$t_{pd}^{\prime }=t_{pd}+\mathrm{GaussianNoise}\times \mathrm{Amplitude}.$$

We anticipate that the effects on *J* from the noisy terms above will exhibit the following characteristics, as shown in Equations (13) and (16), with steps to obtain the results. Using the binomial expansion for ${\left(t_{pd}+\delta \right)}^{4}$,

(12) $${\left(t_{pd}+\delta \right)}^{4}\approx t_{pd}^{4}+4t_{pd}^{3}\delta ,$$

where δ=GaussianNoise×Amplitude.

For the noisy $t_{pd}$ term, the effect on *J* is the following:

(13) $$J^{\prime }\approx \frac{4\left(t_{pd}^{4}+4t_{pd}^{3}\delta \right)}{\Delta^{2}}\left(\frac{1}{U_{d}}+\frac{1}{\Delta }\right).$$

For the noisy Δ term, the effect on *J* is as follows. Using Taylor expansions for the quadratic term,

(14) $${\left(\Delta +\delta \right)}^{2}\approx \Delta^{2}+2\Delta \delta ,$$

where δ=GaussianNoise×Amplitude.

For $\frac{1}{\Delta +\delta }$,

(15) $$\frac{1}{\Delta +\delta }\approx \frac{1}{\Delta }-\frac{\delta }{\Delta^{2}}.$$

Finally, we arrive at the following:

(16) $$J^{\prime }\approx \frac{4t_{pd}^{4}}{\Delta^{2}}\left(1-2\frac{\delta }{\Delta }\right)\left(\frac{1}{U_{d}}+\frac{1}{\Delta }-\frac{\delta }{\Delta^{2}}\right).$$

Due to the size of the on-site bath energy, seen in Equation (5), compared to the impurities’ energies in Equations (6) and (7), it is expected that Δ would not be strongly affected by the noise on the bath-site qubits. Taking into account Equation (4) and that $U_{d}$ would also not be affected by bath-site noise, we can conclude that we expect *J*’s reaction to bath noise to be dominated by the $t_{pd}$ term. On the other hand, for noise on the impurities, Equations (13) and (16) show that competing effects from Δ, $U_{d}$, and $t_{pd}$ are expected to reduce the overall noise. This effect is depicted in both Figure 8 and Figure 9, with noisy bath simulations clearly showing higher error levels for ground-state energy estimations compared to noisy impurity sites.

**Figure 10:**
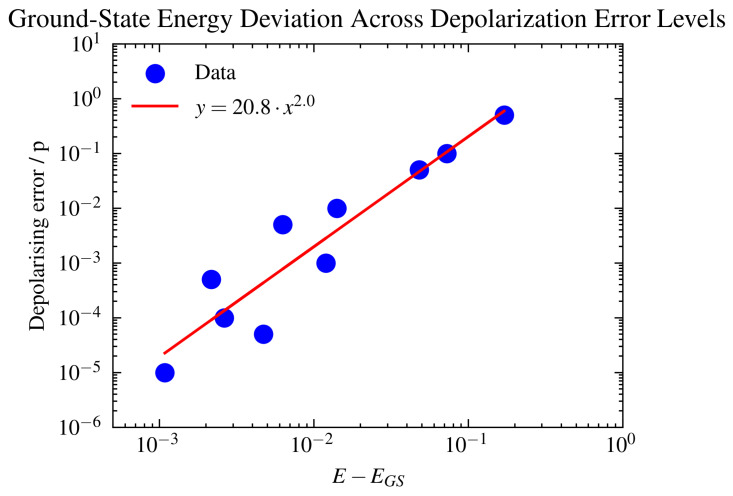
Log–log plot illustrating the difference between the exact ground-state energy, $E_{GS}$, in $E_{h}$ and noisy VQE simulation outcomes across a spectrum of uniform depolarization error rates, ranging from $10^{-1}$ to $10^{-5}$. Each simulation was aligned with the coupling map of IBM_Casablanca to reflect the realistic connectivity between qubits. These ground-state energy estimates were derived from identical 6-qubit VQE simulation configurations employing a generalized UCCSD ansatz, Hamiltonian Config-A, and specified VQE parameters. The observed linear dependence on the log–log scale indicates a systematic and predictable influence of depolarization errors on simulation accuracy with a slope of approximately 2.01.

**Figure 11:**
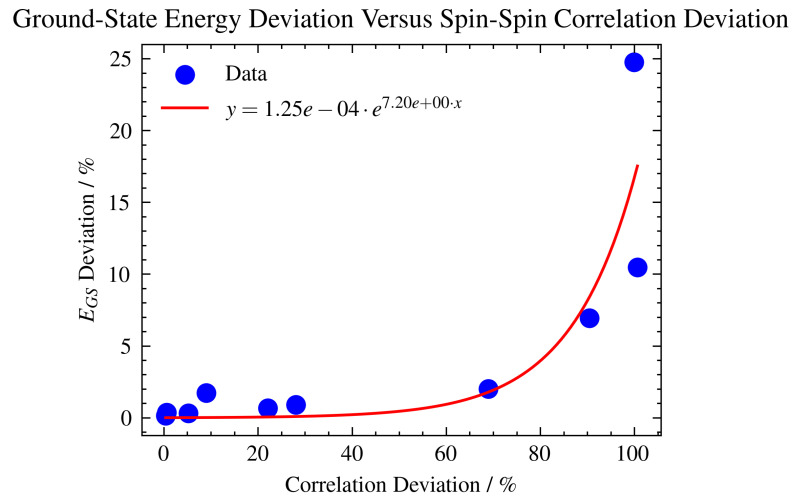
Percentage deviations of noiseless to noisy spin–spin correlation ($\langle S_{z}\left[i\right]*S_{z}\left[j\right]\rangle$) estimates from VQE simulation outcomes across a spectrum of uniform depolarization error rates, ranging from $10^{-1}$ to $10^{-5}$ versus ground-state energy, $E_{GS}$, deviations for the same level of noise. Each simulation was aligned with the coupling map of IBM_Casablanca to reflect the realistic connectivity between qubits. The simulation configurations employ a generalized UCCSD ansatz, Hamiltonian Config-A, and specified VQE parameters. The observed nonlinear relationship in the plot suggests that the correlation results are significantly less stable than the ground-state energy estimates when the same level of noise is present. Exponential fit is added for reference.

In a different approach, in Figure 10 and Figure 11, we investigated the effects of different levels of depolarization error (probability of depolarizing) applied to all 6 qubits on a simulator with a coupling map set to ibm_Casablanca. In real quantum systems, both amplitude damping ($T_{1}$) and phase damping ($T_{2}$) contribute to the overall error in a quantum state. The depolarizing error can be seen as a phenomenological model that encapsulates the net effect of these noises when the detailed behavior of each is not crucial for high-level simulation. It randomly maps the state of a qubit to the maximally mixed state with a certain probability, thus capturing the overall likelihood of a qubit being in an erroneous state due to any noise.

### 2.4. Hardware Topology

Quantum computing holds immense opportunities for accessing new ways of computation, but current NISQ devices suffer from many limitations due to the early stage of the field. In classical computation, the design of the underlying hardware is abstracted away for the user, and operations and measurements do not vary based on the environment of instantaneous runs, producing a deterministic outcome. On the contrary, NISQ devices show large differences between the same Hamiltonian measurements with different topology mappings or even for the same mapping and same hardware but on different days.

**Figure 12:**
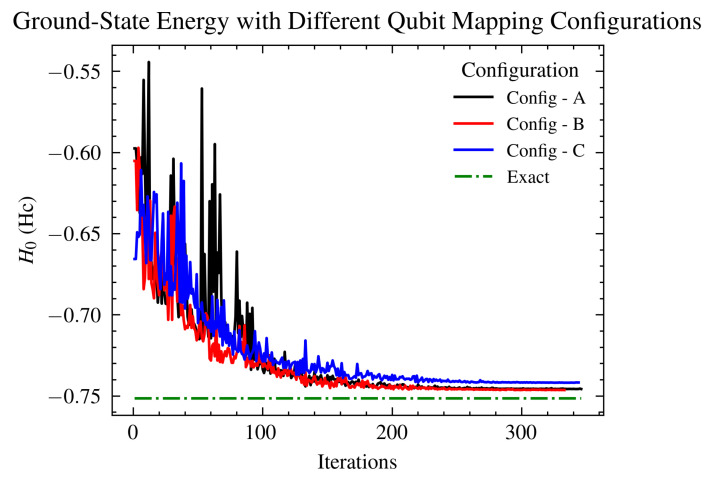
Comparison of ground-state energy estimates in $E_{h}$ derived from different topological mappings of a 6-qubit hemocyanin Hamiltonian onto the qubits. The labels of the configurations correlate to the mappings illustrated in Figure 6, and each simulation employed a generalized UCCSD ansatz with the same coupling map, as shown in Figure 7. Notably, Config−C, which positions the two impurities furthest apart, exhibits the highest error, as anticipated, due to the limited connectivity between the most correlated sites of the Hamiltonian. The other two configurations yield similar results, demonstrating the impact of qubit arrangement on simulation accuracy.

To investigate the level of sensitivity to the topology of the quantum hardware, relevant map shown on Figure 7, we performed ground state energy optimization on different topology mappings. Due to the strong correlation effects in hemocyanin (Hc), we expected to observe differences in the results stemming from incomplete connectivity between the qubits. The topology mappings focused on increasing the physical distance and “quantum” path between the two impurity sites, as these are crucial for representing the modeled Hc accurately. Results are presented in Figure 12.

### 2.5. On-Site Potential

**Figure 13:**
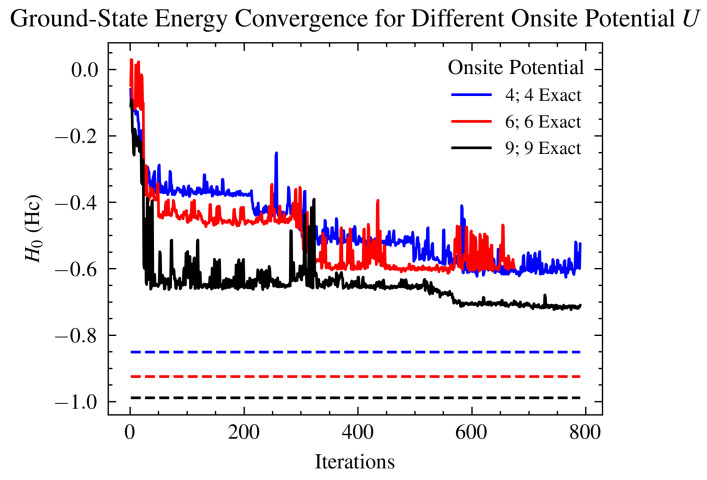
Ground-state energy estimates in $E_{h}$ of the Hc 14-qubit mapped Hamiltonian for different values of the on-site potential *U* in eV. VQE simulation setup using the generalized UCCSD ansatz. As expected, the largest error in the graph is for the U=6eV simulation, which is the suggested point of singlet–triplet transition from [75].

The complex Hc superexchange Cu–O–Cu pathway has been investigated extensively due to its interesting characteristics. Variation in the distance between the mean position of the two copper atoms and the mean position of the two oxygen atoms has shown a singlet-to-triplet transition that occurs at R=0.6, where $R=|\frac{1}{2}\left(r_{\mathrm{CuA}}+r_{\mathrm{CuB}}\right)-\frac{1}{2}\left(r_{O1}+r_{O2}\right)|$ [75]. To investigate this transition, we varied the on-site potential *U*, which accounts for a similar calculation as varying *R*. Figure 13 shows the ground-state convergence of VQE runs for three values of the on-site potential.

### 2.6. Correlation

Conventional many-body methods struggle to correctly predict Hc properties due to the complexity of the superexchange pathway between Cu d-orbitals and intermediate O p-orbitals. The AIM model shows a strong correlation between the two impurity sites, which is difficult to represent in a classical computer simulation but trivial to map using qubit interaction terms. Table 2 represents a comparison between spin–spin correlation measurements for different VQE setups that outline the sensitivity to the topology mapping of the Hamiltonian and to the form of the ansatz. Furthermore, Figure 11 shows the effect of the same levels of depolarization noise on both ground-state energy estimation and impurity spin–spin correlation to assess stability to noise when measuring properties other than energy. Finally, Table 3 presents the type and number of gates of the converged generalized UCCSD ansatz.

## 3. Discussion

Here, we investigated the application of the VQE method for the characterization of a prototypal anti-tumor vaccine metalloprotein (Hc) for the first time on a quantum hardware and simulator, as shown in Figure 3. Varying the configuration of the VQE setup allowed us to understand the dynamics of the simulation and the driving sources of error in depth. Such an analysis proves to be promising for understanding complex entangled systems on quantum devices and their best representation. With the steep improvement in tackling noise on these devices and the growth of the number of qubits and their connectivity on available quantum hardware (Figure 1), it will soon be possible to map larger highly correlated systems directly onto quantum hardware for characterization. A notable example of related work is the recently developed end-to-end quantum simulation workflow by Microsoft Azure Quantum for chemical systems [28]. Leveraging entangled qubits to model highly correlated electron systems on quantum hardware offers a substantial enhancement over traditional computing methods. This is primarily because the underlying principles of quantum mechanics govern both the methodology and systems under investigation, ensuring a more intrinsic and accurate representation.

As illustrated in Figure 3, the noiseless simulator’s VQE run converges to the correct result in fewer than 150 iterations, demonstrating the potential efficacy of simulating quantum phenomena accurately. Conversely, the noisy simulator, incorporating the full spectrum of hardware noise reproduced for the same hardware, struggled to maintain a consistent energy minimization pattern. This challenge primarily stems from the absence of error mitigation techniques, which are crucial in counteracting the inherent noise present in quantum hardware. Furthermore, Figure 3 also highlights the impact of error mitigation in quantum computing simulations, where the hardware execution exhibits a marked convergence, characterized by a significant energy reduction within the first 100 iterations, followed by a more gradual decline. This underscores the importance of error mitigation in enhancing the accuracy and reliability of quantum simulations.

To evaluate the effectiveness of different VQE setups, we conducted simulations using various ansatz types, as shown in Figure 5. The results demonstrated that both the generalized UCCS and generalized UCCSD ansatzes converged to the exact value, with UCCS achieving convergence in fewer iterations (under 50), whereas UCCSD required between 100 to 150 iterations. The latter involves second-order excitations, necessitating a larger pool of ansatz operators and longer circuits, which introduce more noise. However, it captures a broader range of the underlying physical processes. Under conditions of moderate noise, UCCSD is likely to yield more accurate results. Operators count for the generalized UCCSD ansatz with Config−A and can be found in Table 3.

Table 2 presents the spin–spin correlation of impurities measured via VQE simulations, indicating the most accurate results obtained with the spin-conserved UCCSD ansatz, which adheres closely to the physical characteristics of the system. In Figure 5, the simulations for the ground state energy using spin-conserved UCCSD and EfficientSU2 HEA ansatzes both converge to a barren plateau level [74], despite the former closely mirroring true-value impurity correlations. These results underscore the enhancements in system representation when restricting to physically justifiable excitations, though they also highlight potential limitations due to the reduced number of operators in the pool. Future investigations might explore spin-conserved UCC with third-order (or higher-order) excitations to potentially overcome these challenges.

In order to probe the stability of the simulation, we applied T1/T2 thermal relaxation noise to different sets of qubits, observed the effect on the ground state energy, as shown in Figure 8, and further investigated the effect of the depolarization error applied to the whole system, as shown in Figure 10. Due to the lack of error mitigation in these simulations, the addition of noise proves to be critical. Notably, introducing noise to the qubits representing impurity 1 or impurity 2, as expected from their correlation, yielded similar effects on the ground-state energy (refer to Figure 8). Conversely, simulations involving noise at the bath site exhibited a less stable trajectory. These findings underscore the critical role of inter-impurity correlations in the simulation.

Furthermore, investigating the effect of the depolarizing error on the system, as seen in Figure 10 and Figure 11, highlighted a strong quadratic dependence of the ground state energy deviation versus the depolarizing error rate. A slope of approximately 2.01 in a log–log plot of ground-state energy deviation versus depolarizing error rate suggests a specific power–law relationship between the two variables. This means that the ground-state energy deviation varies as the square of the depolarizing error rate, which is defined mathematically as follows:

(17) log(EnergyDeviation)∝2.01·log(DepolarisingError),

(18) $$\mathrm{Energy}\;\mathrm{Deviation}\propto {\left(\mathrm{Depolarising}\;\mathrm{Error}\right)}^{2.01}.$$

A slope of approximately 2 highlights the high sensitivity of the quantum simulation or computation to depolarizing errors. Small increases in error rates cause significant deviations in the calculated ground-state energy, which can severely impact the accuracy and reliability of the measurements. The quadratic relationship emphasizes the compounding effect of errors in quantum systems. This is particularly critical in quantum computations, where maintaining coherence and precision is essential for obtaining correct results. Knowing that the relationship is quadratic allows for more precise modeling and predictions regarding the behavior of a quantum system under noise. This can aid in the design and scaling of quantum algorithms by setting thresholds for acceptable error rates to achieve desired accuracies. Additionally, we presented Figure 11, which illustrates the variations in ground-state energy and spin–spin correlation deviations across different levels of depolarization noise. The percentage error in energy relative to the spin–spin correlation highlights the significant sensitivity of correlation measurements to noise. For instance, 5% error of the noisy to noiseless simulation result for the ground state energy is equivalent to up to 80% error in the correlation estimate. This is an important point to note when considering the characterization of material properties with quantum hardware.

To evaluate the VQE method, one effective approach is to vary the mappings of the impurity and bath sites onto qubits. Given the known spin–spin correlations between impurities, it is expected that positioning their corresponding qubits further apart would lead to less accurate results. Figure 6 illustrates the three different configuration setups used in the experiments, with Config−C having the impurities’ qubits at the greatest distance from each other. The results, presented in Figure 12, confirm this hypothesis: the Config−C simulation converges more slowly to a higher estimate of the ground-state energy, primarily due to noise effects associated with entangling more distantly located qubits.

Each experiment was conducted using the same coupling map to maintain consistent connectivity between qubits, as dictated by the hardware used in the simulations. Over the years, the connectivity of qubits in superconducting quantum hardware has evolved but remains limited, highlighting the significance of qubit mapping in quantum simulations. Our findings highlight the importance of the hardware’s physical layout, which plays a pivotal role in influencing the quality of simulations. Additionally, modern transpilers are essential for effectively adapting simulations to the hardware configuration. Unlike classical computing environments, the layout in quantum computing setups is not yet sufficiently abstracted to ensure that results are independent of qubit ordering, especially in cases of modeling molecules with strong correlations. For an in-depth examination of advancements in qubit connectivity within quantum computing hardware, comprehensive analyses are available through recent scholarly reviews [19,78] and industry developments, particularly from leading entities like IBM [55] and Google [79]. These sources collectively point to the progressive enhancements in qubit design and deployment, significantly influencing the field’s evolution.

In this study, we introduce the first application of a VQE simulation on an AIM model for a highly correlated electron system (Hc) utilizing both quantum hardware and simulators. Adjusting the parameters within the VQE setup facilitated not only the validation of the simulation but also the optimization of the configuration to achieve the best results. The incorporation of noise models, as depicted in Figure 8 and Figure 10, along with the strategic alteration of qubit mappings for the impurities’ sites, as shown in Figure 12, confirmed the alignment between the anticipated trends and the actual convergence to the ground state energy. While the VQE method on contemporary quantum machines is challenged by noise effects, it still showcases a significant capability to surpass traditional quantum chemistry models on classical devices for large systems. This advantage is increasingly amplified through advancements in error mitigation strategies and the ongoing reduction of hardware noise. The Hamiltonians utilized in this study are openly accessible to the community for benchmarking and evaluating enhancements in hardware efficiency.

## 4. Materials and Methods

This investigation utilized a multi-site AIM of the Hamiltonian of Hc molecule developed previously in [75] to measure its properties on IBM [55] and Quantinuum [56] quantum machines with different numbers of qubits and VQE solver setups. The subsections below identify the steps to obtain the model Hamiltonian and describe the VQE algorithm setup.

### 4.1. Molecular Modeling

**Figure 14:**
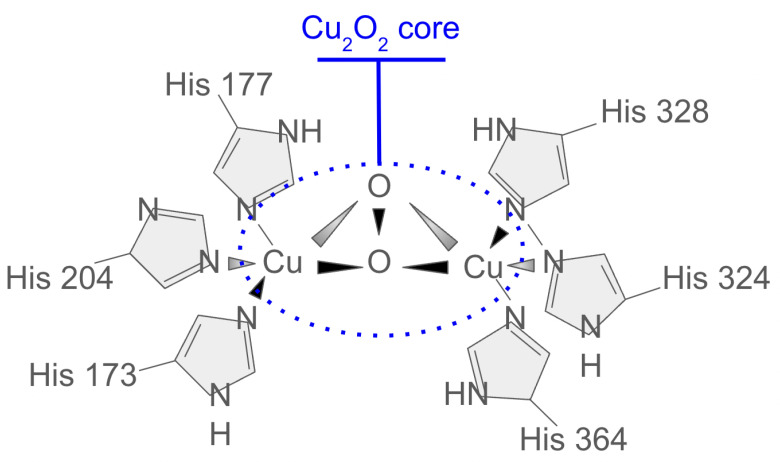
Hemocyanin (oxyHc) $O_{2}$-bound form with an active site $Cu_{2}O_{2}$ core labeled in blue (the $Cu_{2}$ center is a dication (charge not shown)).

Due to the complex character of the binding of oxygen in the formation of oxygenated hemocyanin (oxyHc diagram in Figure 14), most theoretical studies have failed to correctly predict its properties. Here, we follow steps from a previous study on Hc [75] that show agreement with experimental results. The work presents a DFT + DMFT simulation on a 58-atom model of oxyHc. To accurately characterize the superexchange mechanism between Cu_2_ d-orbitals and intermediate O p-orbitals, a non-local (cluster) DMFT is employed. A multi-site AIM captures all correlated sites. which labels the method better as DFT + AIM. A self-consistency cycle over the charge density is performed to ensure a fixed number of electrons.

Anderson impurity model: To solve cluster DMFT [80], we map the hemocyanin core onto an Anderson-impurity model. The AIM can be defined as follows:

(19) $$\begin{array}{l} H_{AIM}=\sum_{mn\sigma }\left(\epsilon_{mn\sigma }^{n}a_{m\sigma }^{†}a_{n\sigma }+\epsilon_{mn\sigma }^{a}a_{m\sigma }^{†}a_{n-\sigma }^{†}+h.c.\right)+\\ \;\;\;\;\;\;\;\;\;\;\;\;\;\;\;\;\;\;\;\;\;\;\;\;\;\;\;\;\;\;\;\;\;\;\;\;\;\;\;\;\;\;\;\;\;\;\;\;\;\;\;\;\;\;\;\;\;\;\sum_{mi\sigma }V_{mi\sigma }\left(a_{m\sigma }^{†}c_{i\sigma }+h.c.\right)+\mu \sum_{i\sigma }c_{i\sigma }^{†}c_{i\sigma }+\sum_{i}U{\hat{n}}_{i↓}{\hat{n}}_{i↑}. \end{array}$$

The fermionic operators $a_{mn}^{†}$ ($a_{mn}$) create (destroy) a particle in the bath, and the fermionic operators $c_{i}^{†}$($c_{i}$) create (destroy) a particle in the cluster of impurities. The indices *m*, *n* run over the bath sites, and the index *i* runs over the impurity sites; the sites of the bath are connected by long-range hopping matrix elements through the particle–hole (particle–particle) channel $\epsilon^{n}$ ($\epsilon^{a}$), the non-correlated sites of the bath are also connected to the correlated impurities by the matrix elements $V_{mi}$, the on-site repulsion at the impurity sites is *U*, and μ is the chemical potential. Here, we map the hemocyanin core onto the AIM by taking the two copper sites as the two impurity sites, with the dioxygen bridge and imidazole ligands acting as the bath. DFT, in conjunction with DMFT, is used to retrieve the AIM parameters. Non-spin-polarized DFT is carried out with Perdew–Burke–Ernzerhof (PBE) functionals, with a non-orthogonal generalized Wannier function (NGWF) [81] gradient threshold of $2\times 10^{-6}$ [82]. A Hubbard U correction of 10 eV is applied to all d-orbitals. DMFT is carried out with U=0.29340, $J_{hund}$ = 0.02939, using the Lanczos impurity solver over 2000 Matsubara frequencies and 800,000 conjugate gradient steps [83]. As such, we obtain the interaction parameters and on-site energies, as presented in Appendix A.

### 4.2. Variational Quantum Eigensolver (VQE) Method

VQE is based on the Rayliegh–Ritz variational principle [35], which finds an upper bound estimate of the expectation value of an observable through the application of a trial wavefunction. In the context of quantum chemistry, this accounts for the optimization of the ground state energy $E_{0}$ of a system with the Hamiltonian $\hat{H}$ and a trial wavefunction $|\psi_{trial}\rangle$ as follows:

(20) $$E_{0}⩽\frac{\langle \psi_{trial}|\hat{H}|\psi_{trial}\rangle }{\langle \psi_{trial}|\psi_{trial}\rangle }.$$

The main part of constructing a robust VQE simulation is choosing a trial wavefunction with an optimal parametrization that can bring the value of the ground state energy estimate to the exact energy within the desired level of precision. In quantum computation, the parametrization step has been the focus of much research due to the vast number of possibilities from hardware efficiency to problem-tailored trial wavefunction, also called ansatz wavefunction. Building an ansatz on a quantum device constitutes the application of quantum gates, which are represented by parametrized unitary operators, labeled U(θ) and applied to *N* qubits, usually initialized in state ${|0\rangle }^{⨂N}$, where θ is a set of parameters that take values in the scope (−π,π].

To represent the Hamiltonian in a form directly measurable on a quantum device, it is transformed into a weighted sum of spin operators using a common mapping technique: Jordan–Wigner transformation (Jordan and Wigner, 1928) [25]. Qiskit does not provide an out-of-the-box mapper between the OpenFermion [84] fermionic Hamiltonian and qiskit Pauli strings representation [55] as a feature for bespoke Hamiltonians; hence, we implemented that step separately. The Hamiltonian is then defined using Pauli strings of the form $\hat{P_{a}}\in {\left\{I,X,Y,Z\right\}}^{⨂N}$, with *N* being the number of qubits used to model the wavefunction:

(21) $$\hat{H}=\sum_{a}^{S}w_{a}\hat{P_{a}}.$$

**Figure 15:**
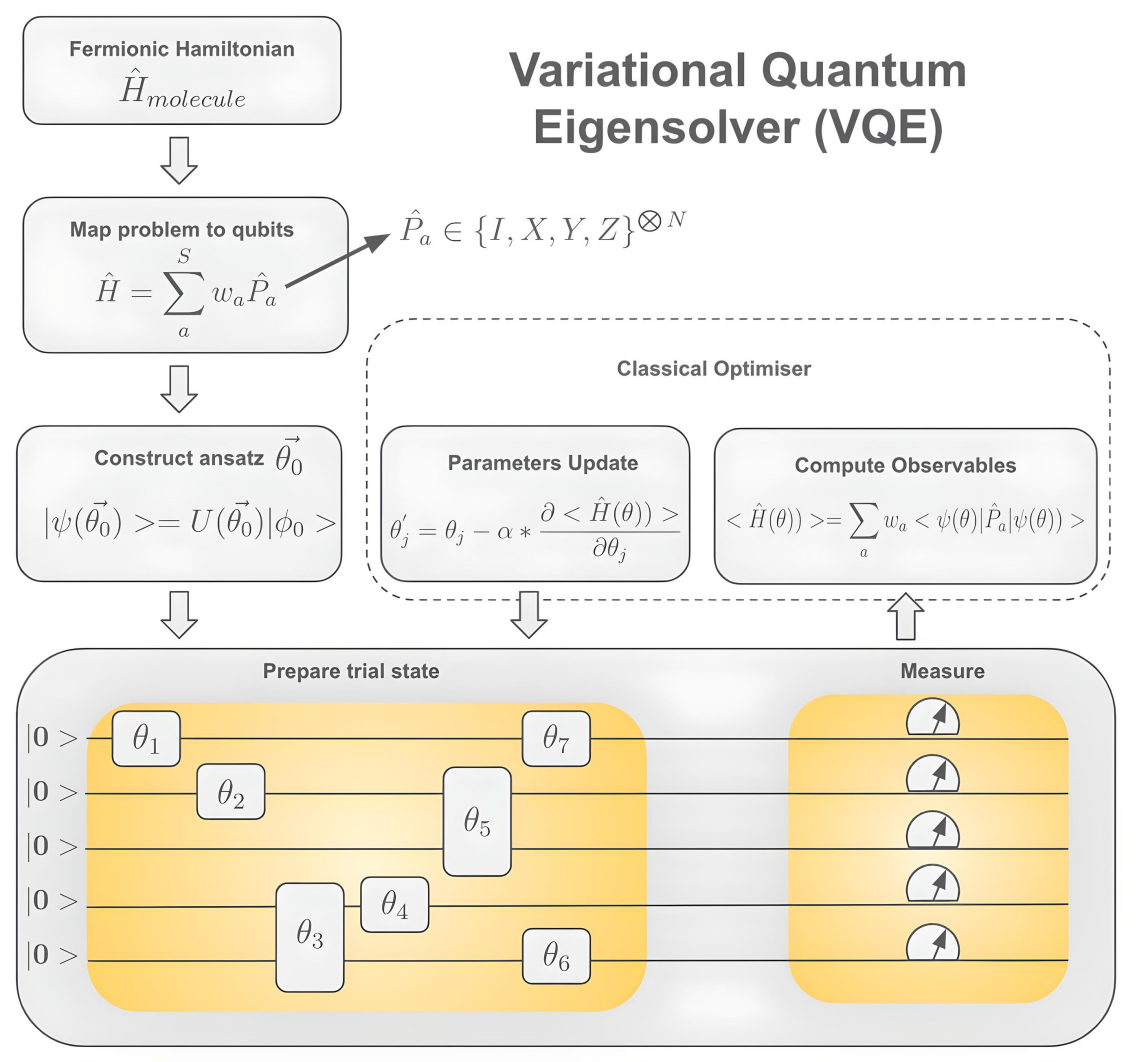
Schematic representation of the variational quantum eigensolver (VQE) method for the minimization of a molecular Hamiltonian by adjusting variational parameters, $\vec{\theta }$. The simulation starts by retrieving the molecular Hamiltonian and mapping it onto a qubit Hamiltonian. Then, the chosen ansatz is applied to the initialized qubit register. After measurements and computation of the observables, new values for the $\vec{\theta^{\prime }}$ are extracted and fed back into the beginning of the loop.

With this representation, Equation (20) using the AIM Hamiltonian, $H_{AIM}$, can be transformed into the following:

(22) $$E_{\mathrm{VQE}}=\min_{\theta }\sum_{a}^{S}w_{a}\langle 0|U^{†}\left(\theta \right){\hat{P}}_{a}U\left(\theta \right)|0\rangle ,$$

where each Pauli string term $\langle 0|U^{†}\left(\theta \right){\hat{P}}_{a}U\left(\theta \right)|0\rangle$ is measured on a quantum device whilst the summation and minimization steps are performed on a conventional computer using a classical optimizer, hence the hybrid nature of quantum computer-based VQE simulations. The complete VQE cycle can be seen in Figure 15.

The design and mapping of the VQE simulation are of great importance to the quality of the results on modern-day NISQ devices; for instance, the depth of the circuits and the number of CNOT gates strongly affect the quantum noise on measurements with shorter circuits, generally leading to more accurate results. The choice of the ansatz is possibly the most important step of the simulation, as shown in Section 2. There are three main categories of ansatz types, and their performance is strongly dependent on the problem at hand. These include the unitary coupled cluster (UCC)-type ansatz [85,86], the hardware-efficient ansatz (HEA) [44,47,87,88,89], and a third type, which is a mixture between UCC and HEA.

The unitary coupled cluster with single and double excitation (UCCSD) ansatz [46,90,91] is a chemistry-inspired ansatz that incorporates knowledge of the underlying quantum system with terms in the ansatz representing specific electronic configurations. The UCCSD trial state is prepared from a reference state $|\phi_{0}\rangle$ by applying exponentiated excitation operators, with $|\phi_{0}\rangle$ commonly chosen as a Hartree–Fock mean-field wave function:

(23) $${\hat{T}}_{1}=\sum_{i=1}^{N_{occ}}\sum_{a=1}^{N_{virt}}t_{i}^{a}{\hat{a}}_{a}^{†}{\hat{a}}_{i},$$

(24) $$\hat{T_{2}}=\sum_{i,j=1}^{N_{occ}}\sum_{a,b=1}^{N_{virt}}t_{ij}^{ab}{\hat{a}}_{a}^{†}{\hat{a}}_{b}^{†}{\hat{a}}_{j}{\hat{a}}_{i}.$$

Here, $\hat{T_{1}}$ and $\hat{T_{2}}$ are the single and double excitation terms, with $\hat{a^{†}}$ and $\hat{a}$ being the creation and annihilation operators, $t_{i}^{a}$ are the single excitation amplitudes, and $t_{ij}^{ab}$ are the double excitation amplitudes. $N_{occ}$ and $N_{virt}$ represent the number of occupied and virtual orbitals, respectively. For UCCS ansatz, only $\hat{T_{1}}$ terms are used, whilst for UCCSD, both terms are applied. A second-degree truncation of the excitations (UCCSD case) forms the following trial wavefunction:

(25) $$\hat{T}=\hat{T_{1}}+\hat{T_{2}},$$

(26) $$U\left(\vec{\theta }\right)=e^{\hat{T}-{\hat{T}}^{†}},$$

(27) $$|\psi_{trial}\rangle =|\psi \left(\vec{\theta }\right)\rangle =e^{\hat{T}-{\hat{T}}^{†}}|\phi_{0}\rangle .$$

**Figure 16:**
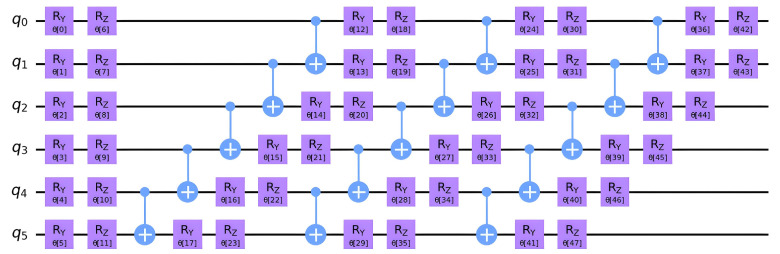
Diagram of EfficientSU2 circuit [92] consisting of layers of single qubit operations spanned by SU(2) and CX entanglements [55]. Hardware efficient ansatz applied on 6 qubits.

The other type of trial wavefunctions is the hardware-efficient ansatz. These ansatzes are constructed from a limited set of gates that are easy to implement on quantum hardware but have no chemical interpretation. An example is the efficientSU2 ansatz [92], which is used in this investigation, as shown in Figure 16.

In this work, we compare the accuracy of VQE simulations on modeled Hc with UCCS, UCCSD, HEA, and bespoke ansatzes. Qiskit implementation allows for adjusting the ansatz parameters, such as specifying the set of excitations or selecting spin-conserving excitations only. The variations in the results between these are presented in Section 2.

## 5. Conclusions

The study demonstrated the potential of the VQE method in characterizing the quantum properties of a prototypal anti-tumor vaccine metalloprotein, Hc, highlighting its capability to model complex entangled systems and capture sensitive properties like spin–spin correlations. In this study, it is crucial to emphasize how the quantum insights gained could impact the protein’s functional role in anti-tumor vaccine development. Specifically, understanding the electron correlations and energy spectra could inform structural optimizations for enhanced immunogenic properties or improved therapeutic efficacy. Future studies could focus on linking these quantum characteristics to measurable biological outcomes, such as the stability of protein and oxygen transport efficiency, thereby solidifying the practical significance of the theoretical findings. Additionally, the multi-site oxygen binding character of Hc could inspire multi-qubit entanglement strategies for quantum hardware design and provide a benchmark for the quality of hardware connectivity and coherence.

Future research could focus on advancing the application of the VQE method by benchmarking on more robust noise mitigation strategies to bridge the gap between simulator and hardware performance. Further research could be performed on incorporating higher-order excitations to better represent complex electron interactions, optimizing qubit mapping and connectivity to enhance simulation efficiency, investigating simulations with a larger number of qubits, and determining the interplay between circuit depth and ansatz pool size. Expanding the scalability of VQE to larger molecular systems and integrating it with hybrid quantum-classical workflows, such as Microsoft Azure Quantum [28], would further broaden its applications. Additionally, collaborations between quantum simulations and experimental chemistry could validate theoretical predictions and provide quality benchmarking. Exploring multi-site correlated systems beyond Hc and focusing on strongly correlated materials could also provide insight into designing robust and scalable quantum hardware.

## Figures and Tables

**Table 1:** Quantum experiments summary: the evolution of quantum chemistry molecule simulations on quantum hardware and emulators from 2013 to the present. The recent entries, highlighted in the table’s final three rows in blue, demonstrate consistency in the size of qubits employed but also an expansion to encompass larger molecular systems. Data in the table are based on [39].

Year	Qubits	Systems	Methods	Platform	Hardware Max Qubits	Company
2013	2	HeH+	VQE-UCC	Silicon Photonic	2	In-house [40]
2015	2	HeH+	VQE-UCC	Trapped ion	2	IonQ [41]
2015	2	H_2_	VQE-UCC	Superconducting	9	Google [42]
2016	2	H_2_, $H_{3}^{+}$, H_4_	IPEA, VQE-UCC	Silicon photonic	2	In-house [43]
2017	6	H_2_, LiH, BeH_2_, Heisenberg model	Hardware-efficient VQE	Superconducting	20	IBM [44]
2017	2	H_2_ (excited states)	Hardware-specific VQE	Superconducting	20	UC Berkeley [45]
2018	3	H_2_, LiH	VQE-UCC	Trapped-ion	11	Honeywell [46]
2018	4	Quantum magnetism, H_2_, LiH	Hardware-efficient VQE	Superconducting	20	IBM [47]
2018	4	H_2_, LiH	Qubit CC	Superconducting	53	Google [48]
2019	2	H_2_O	QPE	NMR	2	In-house [49]
2019	4	H_2_O	VQE-UCC	Trapped-ion	11	IonQ [26]
2019	4	NaH, RbH, KH	Hardware-efficient VQE(-UCC)	Superconducting	53	ORNL [8]
2019	2	Lithium superoxide dimer	VQE-UCC	Superconducting	53	IBM [50]
2019	3	H_3_	VQE-UCC	Superconducting	53	Google [51]
2020	12	H_6_, H_8_, H_10_, H_12_, HNNH	VQE-HF	Superconducting	53	Google [48]
2020	2	PSPCz, 2F-PSPCz, 4F-PSPCz	qEOM-VQE, VQD	Superconducting	127	IBM [52]
2022	28	C_2_H_4_	Point Symmetry	Emulator	433	Quantum Emulation Group [53]
2022	72	${I_{r}}^{III}$ Complexes	Point Symmetry	Emulator	1000+	Quantum Emulation Group [54]
2024	6	oxyHc (58-atom model)	VQE-UCC	Superconducting	7	IBM [55]
2024	6	oxyHc (58-atom model)	VQE-UCC	Superconducting	20	Quantinuum [56]
2024	14	oxyHc (58-atom model)	VQE-UCC	Emulator	50+	IBM [55]

**Table 2:** Hemocyanin AIM impurity spin–spin correlation measurements and exact values [75] (6 Qubits).

Topology	VQE Setup	$\langle S_{z}\left[i\right]\cdot S_{z}\left[j\right]\rangle$
	Exact	−0.307
Config-A	Spin-conserved UCCSD	−0.314
Config-A	UCCS	−0.248
Config-A	Generalised UCCSD	−0.169
Config-B	Generalised UCCSD	−0.018
Config-C	Generalised UCCSD	−0.078

**Table 3:** Simulation of hemocyanin using the generalized UCCSD ansatz with Config−A mapping resulted in energy estimates closest to the exact value based on the following operator counts.

Operator	Count
U2 Gate	288
CNOT Gate (Controlled-NOT)	280
U1 Gate	184
U Gate	4

**Figure A1:**
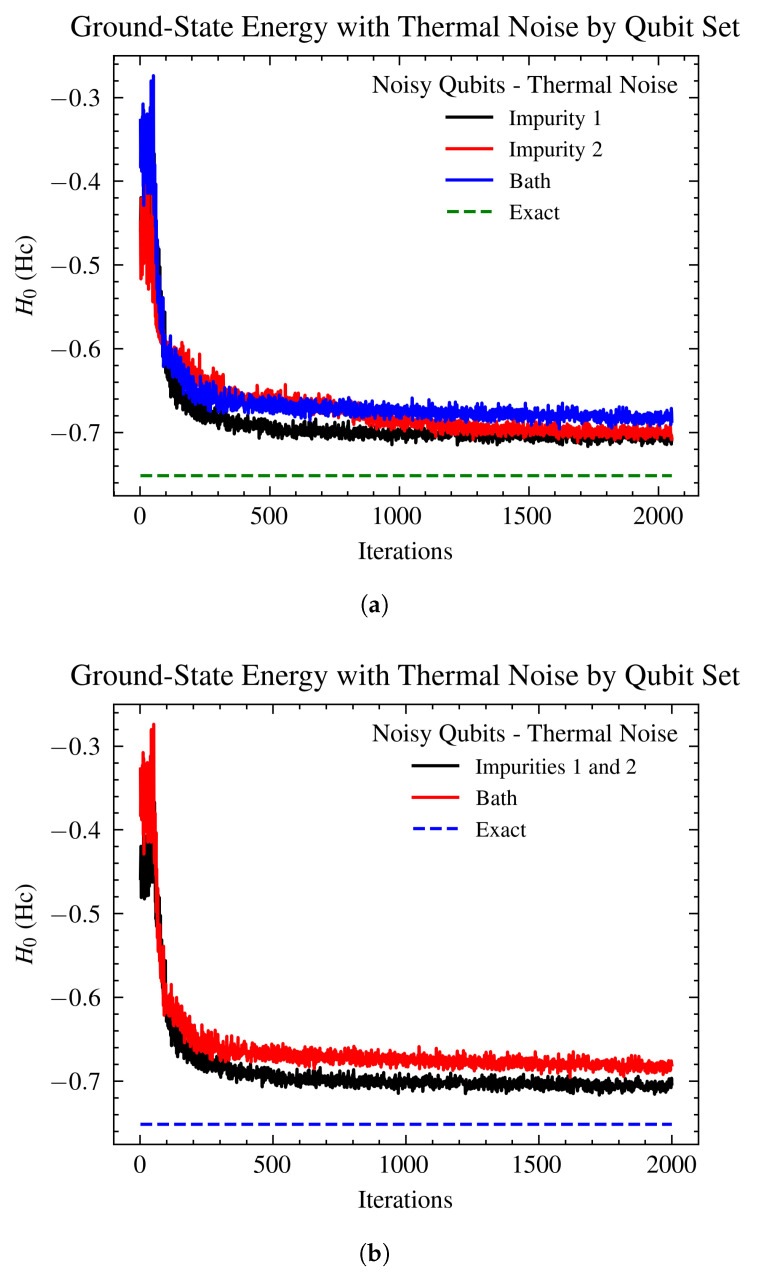
(**a**) Comparison of ground-state energy estimates for 6-qubit VQE simulations (generalized UCCSD ansatz and Hamiltonian Config−A) under halved $T_{1}/T_{2}$ thermal relaxation noise levels. Noise is selectively applied to impurity 1 (qubits 0,1), impurity 2 (qubits 2,3), and bath (qubits 4,5) subsets. Consistent with previous findings, noise on the bath sites leads to more pronounced performance degradation than noise on impurity sites. (**b**) Additional comparison of ground-state energy estimates for 6-qubit VQE simulations (generalized UCCSD ansatz and Hamiltonian Config−A) under $T_{1}/T_{2}$ thermal relaxation noise. Noise is selectively applied to qubits representing impurity 1 (qubits 0,1), impurity 2 (qubits 2,3), and bath (qubits 4,5). As before, the similarity of results between noise applied to either impurity set and the relative vulnerability of the bath sites is evident.

## Data Availability

The code developed throughout this investigation is available at the following GitHub Repository: https://github.com/elenachachkarova/hemocyanin-vqe.

## Abbreviations

    The following abbreviations are used in this manuscript:

AIM	Anderson Impurity Model
CNOT	Controlled-NOT
DFT	Density Functional Theory
DMFT	Dynamical Mean Field Theory
Hc	Hemocyanin
HEA	Hardware-Efficient Ansatz
KLH	Keyhole Limpet Hemocyanin
NGWF	Non-orthogonal Generalized Wannier Function
NISQ	Noisy Intermediate-Scale Quantum
NMR	Nuclear Magnetic Resonance
PBE	Perdew–Burke–Ernzerhof
QASM	Quantum Assembly
qEOM	Quantum Equation-of-Motion
QPE	Quantum Phase Estimation
QPU	Quantum Processing Unit
SPSA	Simultaneous Perturbation Stochastic Approximation
UCC	Unitary Coupled Cluster
UCCS	Unitary Coupled Cluster with singles
UCCSD	Unitary Coupled Cluster with singles and doubles
VQE	Variational Quantum Eigensolver
VQD	Variational Quantum Deflation

## Appendix A

Below are the constructed Hamiltonians used in the VQE solver in their transformed Pauli string form, corresponding to the number of qubits and chosen on-site potential (U). These details complement the descriptions and methodologies discussed in the main manuscript, offering deeper insight into the specific Hamiltonians studied.

6 Qubit

(-0.4640485702054111+0j) [] +

(-0.0332912087832737+0j) [X0 Z1 Z2 Z3 X4] +

(-0.0332912087832737+0j) [Y0 Z1 Z2 Z3 Y4] +

(0.06874150000000001+0j) [Z0] +

(0.07335+0j) [Z0 Z1] +

(-0.0332912087832737+0j) [X1 Z2 Z3 Z4 X5] +

(-0.0332912087832737+0j) [Y1 Z2 Z3 Z4 Y5] +

(0.06874150000000001+0j) [Z1] +

(0.0245514029353716+0j) [X2 Z3 X4] +

(0.0245514029353716+0j) [Y2 Z3 Y4] +

(0.058285350000000014+0j) [Z2] +

(0.07335+0j) [Z2 Z3] +

(0.0245514029353716+0j) [X3 Z4 X5] +

(0.0245514029353716+0j) [Y3 Z4 Y5] +

(0.058285350000000014+0j) [Z3] +

(0.0316474351027055+0j) [Z4] +

(0.0316474351027055+0j) [Z5]

8 Qubit

(-0.41926934879169153+0j) [] +

(0.0169273012830259+0j) [X0 Z1 Z2 Z3 X4] +

(-0.0124834455965616+0j) [X0 Z1 Z2 Z3 Z4 Z5 X6] +

(0.0169273012830259+0j) [Y0 Z1 Z2 Z3 Y4] +

(-0.0124834455965616+0j) [Y0 Z1 Z2 Z3 Z4 Z5 Y6] +

(0.06874150000000001+0j) [Z0] +

(0.07335+0j) [Z0 Z1] +

(0.0169273012830259+0j) [X1 Z2 Z3 Z4 X5] +

(-0.0124834455965616+0j) [X1 Z2 Z3 Z4 Z5 Z6 X7] +

(0.0169273012830259+0j) [Y1 Z2 Z3 Z4 Y5] +

(-0.0124834455965616+0j) [Y1 Z2 Z3 Z4 Z5 Z6 Y7] +

(0.06874150000000001+0j) [Z1] +

(-0.0286665490477019+0j) [X2 Z3 X4] +

(0.02114083628193125+0j) [X2 Z3 Z4 Z5 X6] +

(-0.0286665490477019+0j) [Y2 Z3 Y4] +

(0.02114083628193125+0j) [Y2 Z3 Z4 Z5 Y6] +

(0.058285350000000014+0j) [Z2] +

(0.07335+0j) [Z2 Z3] +

(-0.0286665490477019+0j) [X3 Z4 X5] +

(0.02114083628193125+0j) [X3 Z4 Z5 Z6 X7] +

(-0.0286665490477019+0j) [Y3 Z4 Y5] +

(0.02114083628193125+0j) [Y3 Z4 Z5 Z6 Y7] +

(0.058285350000000014+0j) [Z3] +

(0.0236589565634185+0j) [X4 Z5 X6] +

(0.0236589565634185+0j) [Y4 Z5 Y6] +

(-0.00841924169912505+0j) [Z4] +

(0.0236589565634185+0j) [X5 Z6 X7] +

(0.0236589565634185+0j) [Y5 Z6 Y7] +

(-0.00841924169912505+0j) [Z5] +

(0.0176770660949708+0j) [Z6] +

(0.0176770660949708+0j) [Z7]

14 Qubit

(-0.9203822755004079+0j) [] +

(-0.0102330968552639+0j) [X0 Z1 Z2 Z3 X4] +

(-0.0058645309558+0j) [X0 Z1 Z2 Z3 Z4 Z5 X6] +

(-0.0232723792309368+0j) [X0 Z1 Z2 Z3 Z4 Z5 Z6 Z7 X8] +

(0.0285112773771463+0j) [X0 Z1 Z2 Z3 Z4 Z5 Z6 Z7 Z8 Z9 X10] +

(0.03368107911672325+0j) [X0 Z1 Z2 Z3 Z4 Z5 Z6 Z7 Z8 Z9 Z10 Z11 X12] +

(-0.0102330968552639+0j) [Y0 Z1 Z2 Z3 Y4] +

(-0.0058645309558+0j) [Y0 Z1 Z2 Z3 Z4 Z5 Y6] +

(-0.0232723792309368+0j) [Y0 Z1 Z2 Z3 Z4 Z5 Z6 Z7 Y8] +

(0.0285112773771463+0j) [Y0 Z1 Z2 Z3 Z4 Z5 Z6 Z7 Z8 Z9 Y10] +

(0.03368107911672325+0j) [Y0 Z1 Z2 Z3 Z4 Z5 Z6 Z7 Z8 Z9 Z10 Z11 Y12] +

(0.06874150000000001+0j) [Z0] +

(0.07335+0j) [Z0 Z1] +

(-0.0102330968552639+0j) [X1 Z2 Z3 Z4 X5] +

(-0.0058645309558+0j) [X1 Z2 Z3 Z4 Z5 Z6 X7] +

(-0.0232723792309368+0j) [X1 Z2 Z3 Z4 Z5 Z6 Z7 Z8 X9] +

(0.0285112773771463+0j) [X1 Z2 Z3 Z4 Z5 Z6 Z7 Z8 Z9 Z10 X11] +

(0.03368107911672325+0j) [X1 Z2 Z3 Z4 Z5 Z6 Z7 Z8 Z9 Z10 Z11 Z12 X13] +

(-0.0102330968552639+0j) [Y1 Z2 Z3 Z4 Y5] +

(-0.0058645309558+0j) [Y1 Z2 Z3 Z4 Z5 Z6 Y7] +

(-0.0232723792309368+0j) [Y1 Z2 Z3 Z4 Z5 Z6 Z7 Z8 Y9] +

(0.0285112773771463+0j) [Y1 Z2 Z3 Z4 Z5 Z6 Z7 Z8 Z9 Z10 Y11] +

(0.03368107911672325+0j) [Y1 Z2 Z3 Z4 Z5 Z6 Z7 Z8 Z9 Z10 Z11 Z12 Y13] +

(0.06874150000000001+0j) [Z1] +

(-0.01395767436142885+0j) [X2 Z3 X4] +

(0.02042556305865555+0j) [X2 Z3 Z4 Z5 X6] +

(-0.0342747841006893+0j) [X2 Z3 Z4 Z5 Z6 Z7 X8] +

(-0.0194319517229672+0j) [X2 Z3 Z4 Z5 Z6 Z7 Z8 Z9 X10] +

(-0.0051669611836042+0j) [X2 Z3 Z4 Z5 Z6 Z7 Z8 Z9 Z10 Z11 X12] +

(-0.01395767436142885+0j) [Y2 Z3 Y4] +

(0.02042556305865555+0j) [Y2 Z3 Z4 Z5 Y6] +

(-0.0342747841006893+0j) [Y2 Z3 Z4 Z5 Z6 Z7 Y8] +

(-0.0194319517229672+0j) [Y2 Z3 Z4 Z5 Z6 Z7 Z8 Z9 Y10] +

(-0.0051669611836042+0j) [Y2 Z3 Z4 Z5 Z6 Z7 Z8 Z9 Z10 Z11 Y12] +

(0.058285350000000014+0j) [Z2] +

(0.07335+0j) [Z2 Z3] +

(-0.01395767436142885+0j) [X3 Z4 X5] +

(0.02042556305865555+0j) [X3 Z4 Z5 Z6 X7] +

(-0.0342747841006893+0j) [X3 Z4 Z5 Z6 Z7 Z8 X9] +

(-0.0194319517229672+0j) [X3 Z4 Z5 Z6 Z7 Z8 Z9 Z10 X11] +

(-0.0051669611836042+0j) [X3 Z4 Z5 Z6 Z7 Z8 Z9 Z10 Z11 Z12 X13] +

(-0.01395767436142885+0j) [Y3 Z4 Y5] +

(0.02042556305865555+0j) [Y3 Z4 Z5 Z6 Y7] +

(-0.0342747841006893+0j) [Y3 Z4 Z5 Z6 Z7 Z8 Y9] +

(-0.0194319517229672+0j) [Y3 Z4 Z5 Z6 Z7 Z8 Z9 Z10 Y11] +

(-0.0051669611836042+0j) [Y3 Z4 Z5 Z6 Z7 Z8 Z9 Z10 Z11 Z12 Y13] +

(0.058285350000000014+0j) [Z3] +

(0.00800357635717085+0j) [X4 Z5 X6] +

(-0.04725338824796345+0j) [X4 Z5 Z6 Z7 X8] +

(0.00696745171857815+0j) [X4 Z5 Z6 Z7 Z8 Z9 X10] +

(0.0036965854307191+0j) [X4 Z5 Z6 Z7 Z8 Z9 Z10 Z11 X12] +

(0.00800357635717085+0j) [Y4 Z5 Y6] +

(-0.04725338824796345+0j) [Y4 Z5 Z6 Z7 Y8] +

(0.00696745171857815+0j) [Y4 Z5 Z6 Z7 Z8 Z9 Y10] +

(0.0036965854307191+0j) [Y4 Z5 Z6 Z7 Z8 Z9 Z10 Z11 Y12] +

(0.05178138173911655+0j) [Z4] +

(0.00800357635717085+0j) [X5 Z6 X7] +

(-0.04725338824796345+0j) [X5 Z6 Z7 Z8 X9] +

(0.00696745171857815+0j) [X5 Z6 Z7 Z8 Z9 Z10 X11] +

(0.0036965854307191+0j) [X5 Z6 Z7 Z8 Z9 Z10 Z11 Z12 X13] +

(0.00800357635717085+0j) [Y5 Z6 Y7] +

(-0.04725338824796345+0j) [Y5 Z6 Z7 Z8 Y9] +

(0.00696745171857815+0j) [Y5 Z6 Z7 Z8 Z9 Z10 Y11] +

(0.0036965854307191+0j) [Y5 Z6 Z7 Z8 Z9 Z10 Z11 Z12 Y13] +

(0.05178138173911655+0j) [Z5] +

(0.0043850975092616+0j) [X6 Z7 X8] +

(-0.01487110793047115+0j) [X6 Z7 Z8 Z9 X10] +

(-0.02364586870809315+0j) [X6 Z7 Z8 Z9 Z10 Z11 X12] +

(0.0043850975092616+0j) [Y6 Z7 Y8] +

(-0.01487110793047115+0j) [Y6 Z7 Z8 Z9 Y10] +

(-0.02364586870809315+0j) [Y6 Z7 Z8 Z9 Z10 Z11 Y12] +

(0.03514668312536425+0j) [Z6] +

(0.0043850975092616+0j) [X7 Z8 X9] +

(-0.01487110793047115+0j) [X7 Z8 Z9 Z10 X11] +

(-0.02364586870809315+0j) [X7 Z8 Z9 Z10 Z11 Z12 X13] +

(0.0043850975092616+0j) [Y7 Z8 Y9] +

(-0.01487110793047115+0j) [Y7 Z8 Z9 Z10 Y11] +

(-0.02364586870809315+0j) [Y7 Z8 Z9 Z10 Z11 Z12 Y13] +

(0.03514668312536425+0j) [Z7] +

(-0.01037904738535395+0j) [X8 Z9 X10] +

(0.00386000942055545+0j) [X8 Z9 Z10 Z11 X12] +

(-0.01037904738535395+0j) [Y8 Z9 Y10] +

(0.00386000942055545+0j) [Y8 Z9 Z10 Z11 Y12] +

(0.07645310066235694+0j) [Z8] +

(-0.01037904738535395+0j) [X9 Z10 X11] +

(0.00386000942055545+0j) [X9 Z10 Z11 Z12 X13] +

(-0.01037904738535395+0j) [Y9 Z10 Y11] +

(0.00386000942055545+0j) [Y9 Z10 Z11 Z12 Y13] +

(0.07645310066235694+0j) [Z9] +

(-0.0472194011845761+0j) [X10 Z11 X12] +

(-0.0472194011845761+0j) [Y10 Z11 Y12] +

(0.0472879363412088+0j) [Z10] +

(-0.0472194011845761+0j) [X11 Z12 X13] +

(-0.0472194011845761+0j) [Y11 Z12 Y13] +

(0.0472879363412088+0j) [Z11] +

(0.0491451858821573+0j) [Z12] +

(0.0491451858821573+0j) [Z13]

## Appendix B

Figure A1 presents additional comparisons of ground-state energy estimates under different thermal relaxation noise levels. By examining these conditions, the reader can better understand how reduced noise intensities or selective noise targeting of certain qubit subsets affect VQE simulation outcomes.
